# Human melanoma immunotherapy using tumor antigen-specific T cells generated in humanized mice

**DOI:** 10.18632/oncotarget.7044

**Published:** 2016-01-27

**Authors:** Zheng Hu, Jinxing Xia, Wei Fan, Jennifer Wargo, Yong-Guang Yang

**Affiliations:** ^1^ The First Bethune Hospital and Institute of Immunology, Jilin University, Changchun, China; ^2^ Columbia Center for Translational Immunology, Department of Medicine, Columbia University College of Physicians and Surgeons, New York, NY, USA; ^3^ Department of Surgery, Massachusetts General Hospital, Harvard Medical School, Boston, MA, USA

**Keywords:** animal model, immunotherapy, melanoma, TCR, T cells, Immunology and Microbiology Section, Immune response, Immunity

## Abstract

A major factor hindering the exploration of adoptive immunotherapy in preclinical settings is the limited availability of tumor-reactive human T cells. Here we developed a humanized mouse model that permits large-scale production of human T cells expressing the engineered melanoma antigen MART-1-specific TCR. Humanized mice, made by transplantation of human fetal thymic tissue and CD34^+^ cells virally-transduced with HLA class I-restricted melanoma antigen (MART-1)-specific TCR gene, showed efficient development of MART-1-TCR^+^ human T cells with predominantly CD8^+^ cells. Importantly, MART-1-TCR^+^CD8^+^ T cells developing in these mice were capable of mounting antigen-specific responses *in vivo*, as evidenced by their proliferation, phenotypic conversion and IFN-γ production following MART-1 peptide immunization. Moreover, these MART-1-TCR^+^CD8^+^ T cells mediated efficient killing of melanoma cells in an HLA/antigen-dependent manner. Adoptive transfer of *in vitro* expanded MART-1-TCR^+^CD8^+^ T cells induced potent antitumor responses that were further enhanced by IL-15 treatment in melanoma-bearing recipients. Finally, a short incubation of MART-1-specific T cells with rapamycin acted synergistically with IL-15, leading to significantly improved tumor-free survival in recipients with metastatic melanoma. These data demonstrate the practicality of using humanized mice to produce potentially unlimited source of tumor-specific human T cells for experimental and preclinical exploration of cancer immunotherapy. This study also suggests that pretreatment of tumor-reactive T cells with rapamycin in combination with IL-15 administration may be a novel strategy to improve the efficacy of adoptive T cell therapy.

## INTRODUCTION

Infusion of *in vitro* expanded autologous tumor-infiltrating lymphocytes (TILs) following lymphodepletion has been shown to result in objective tumor regression in up to 70% of patients with metastatic melanoma, and almost a quarter of the treated patients achieved durable complete remission [1]. However, it is not always possible to obtain TILs with anti-melanoma activity and there has been limited success in obtaining TILs in other cancers. Thus, much effort has been devoted to develop efficient means of producing CTLs with antitumor activity. In addition, melanoma frequently relapses in the patients after a period of remission [1], and the relapse was found to be associated with a tumor immunosuppressive microenvironment that inhibits T cell function [2]. Emerging evidence indicates that the tumor-induced inhibition of T cell activation is largely attributed to the recruitment of regulatory T cells (Tregs) into the tumor and upregulation of immune inhibitory pathway signaling, which are both driven by T cell immune responses [3, 4]. These studies imply that, for achieving the desired therapeutic effects of adoptive immunotherapy, it is important to develop effective approaches overcoming these immunosuppressive pathways. However, such studies have mostly been performed in mice, and the limited availability of tumor-reactive human CTLs that resemble those from patients is one of the key impeding factors.

It has been shown first in mice [5, 6], and more recently in humans [7] that T cells expressing the transgenic TCR can be generated by introducing TCR genes into hematopoietic stem cells. We have previously shown that transplantation of human fetal thymus tissue (FTHY; under kidney capsule) and CD34^+^ fetal liver cells (FLCs; i.v.) in immunodeficient mice leads to the development of human lymphohematopoietic cells including T, B and dendritic cells, and the formation of secondary lymphoid organs consisting of human lymphohematopoietic cells [8-10]. Here, we investigate the possibility of using this humanized mouse (hu-mouse) model to generate melanoma antigen (MART-1)-specific human T cells for translational studies of adoptive cancer immunotherapies. We show that MART-1-specific human T cells can be generated efficiently in hu-mice made of CD34^+^ FLCs that were transduced with lentiviruses containing MART-1-specific TCR gene. Importantly, MART-1-specific human T cells developed in hu-mice are functional and capable of killing melanoma cells in an HLA/peptide-dependent manner. Furthermore, using hu-mouse-derived melanoma antigen-specific human T cells, we demonstrate that pretreatment of the T cells with rapamycin can significantly enhance the antitumor activity of adoptive T cell therapy in IL-15-treatted recipients.

## RESULTS

### Development of melanoma antigen MART-1-specific human T cells in humanized mice made of TCR engineered CD34^+^ cells

**Figure 1:**
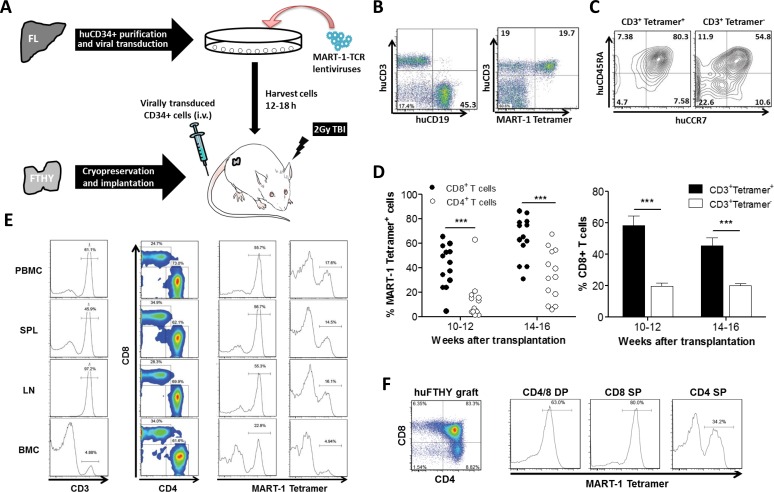
Generation of MART-1-specific T cells in humanized mice made by transplantation of human FTHY and TCR-engineered CD34^**+**^ FLCs **A.** Schematic preparation of the humanized mouse model with human T cells expressing transgenic TCR specific for MART-1. **B.** Representative flow cytometric profiles showing reconstitution of human T and B cells (left) and MART-1 TCR^+^ T cells (right) in PBMCs of humanized mice. **C.** FACS analysis of MART-1 TCR^+^ and MART-1 TCR^−^ T cells for CD45RA and CCR7 expression. **D.** Shown are percentages of MART-1 TCR^+^ T cells in CD8^+^ and CD4^+^ T cell compartments (left) and percentages (mean ± SEMs; *n* = 13) of CD8^+^ T cells in MART-1 TCR^+^ and MART-1 TCR^−^ T cell population (right). **E.** FACS assessment of MART-1 TCR^+^ T cells in CD8^+^ and CD4^+^ T cells from the indicated tissues at week 22. **F.** FACS assessment of MART-1 TCR^+^ T cells in CD4^+^CD8^+^ (DP), CD8SP and CD4SP human thymocytes. ***, *p* < 0.001.

**Figure 2:**
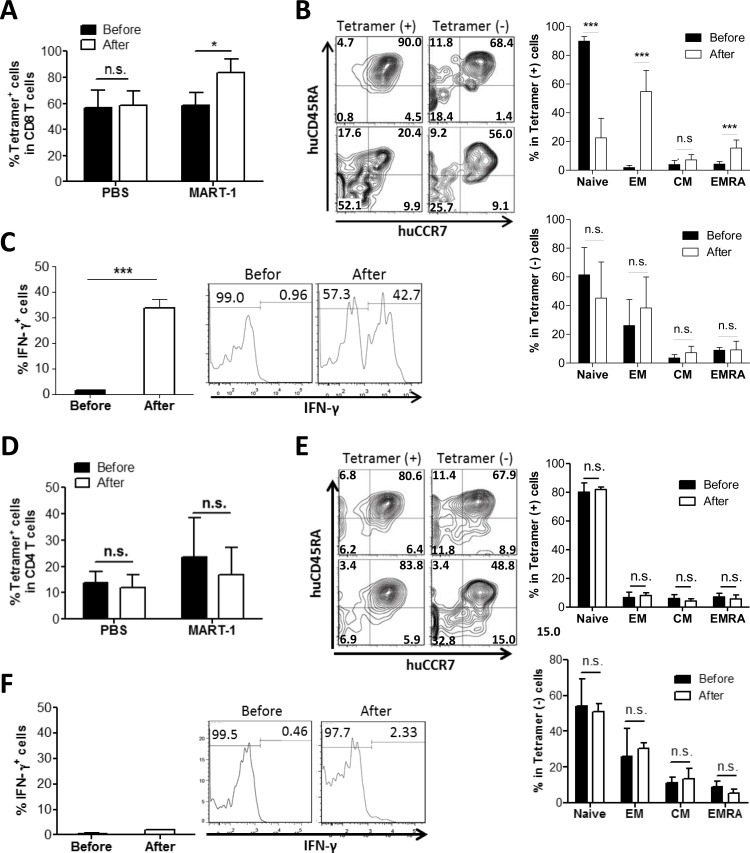
MART-1 TCR**+** CD8, but not CD4, T cells show antigen-specific responses in humanized mice following MART-1 peptide immunization Hu-mice were immunized with MART-1 peptide or PBS as control 15 weeks after humanization, and CD8^+^ **A.**-**C.** and CD4^+^ **D.**-**F.** T cell responses were assessed by flow cytometry 1 week prior to (before) and 3 weeks after immunization. **A.** and **D.** Percentages of tetramer^+^ cells in peripheral blood CD8^+^ **A.** and CD4^+^ **D.** T cells (mean±SEM; *n* = 3). **B.** and **E.** Expression of CD45RA and CCR7 on tetramer^+^ *vs*. tetramer^−^ CD8^+^ **B.** and CD4^+^ **E.** T cells (mean ± SEMs; *n* = 7). *Left*, representative staining profiles of the cells prepared before (top) and after (bottom) immunization; *Right*, percentages of T cell subsets in tetramer^+^ (top) and tetramer^−^ (bottom) T cells prepared before and after immunization. Naïve, CD45RA^+^CCR7^+^ naïve T cells; EM, CD45RA^−^CCR7^−^ effector memory T cells; CM, CD45RA^−^CCR7^+^ central memory T cells; EM/RA, CD45RA^+^CCR7^−^ effector memory T cells. **C.** and **F.** Percentages of IFN-γ producing tetramer^+^ CD8^+^ **C.** and CD4^+^ **F.** T cells prepared before and after immunization (mean ± SEMs; *n* = 5). *, *p* < 0.05; **, *p* < 0.01; ***, *p* < 0.001; n.s., not significant.

A lentiviral vector encoding HLA-A*0201-restricted TCR (DMF5 clone) [11] specific for melanoma-associated antigen recognized by T cell-1 (MART-1) was used to engineer CD34^+^ FLCs. The hu-mice were made by intravenous injection of TCR-engineered HLA-A*0201^+^ CD34^+^ FLCs into NOD.Cg-Prkdc^scid^ Il2rg^tm1Wjl^/SzJ (NSG) mice grafted with cryopreserved-thawed autologous FTHY (Figure 1A). We have shown that the use of cryopreserved-thawed FTHY may improve T cell development from virally-transduced CD34^+^ cells by eliminating preexisting T cell progenitors in the FTHY graft (Hu Z, Xia J, Yang YG. Unpublished data). In hu-mice that received HLA-A*0201^+^ FTHY and CD34^+^ FLCs transduced with lentiviral vectors containing MART-1-specific TCR gene, high levels of human T and B cell reconstitution were detected (Figure S1) and among human CD3^+^ T cells, a significant proportion was found to express MART-1-specific TCR, as identified by HLA-A2/MART-1 tetramer staining (Figure 1B). The majority of tetramer^+^ T cells had a naïve phenotype as shown by expression of CD45RA and CCR7 (Figure 1C). In concordance with the role of CD8 in recognition of MHC class I-restricted antigens, CD8^+^ T cells consisted of a significantly larger number of tetramer^+^ cells than CD4^+^ T cells (especially at the early time), and the percentage of CD8^+^ T cells in CD3^+^tetramer^+^ cells was significantly higher than found in CD3^+^tetramer^−^ T cells (Figure 1D and 1E). All mice had at least more than a million (ranging from 1.1×10^6^ and 12×10^6^) of tetramer^+^ CD8^+^ T cells in the spleen, and a significant increase was seen in hu-mice receiving MART-1 peptide immunization (Table S1; also see Figure 2 below). Furthermore, most CD8 single positive (SP) and CD4^+^CD8^+^ double positive (DP) thymocytes expressed MART-1-specific TCR (Figure 1F). Although the frequency was lower, peripheral human CD4^+^ T cells and CD4SP thymocytes also contained a significant proportion of tetramer^+^ cells (with relatively lower levels of MART-1-TCR expression; Figure S2) in almost all mice examined (Figure 1D-1F).

### Tetramer^+^ CD8+, but not CD4^+^, T cells showed efficient *in vivo* responses following peptide immunization

Next we questioned if the tetramer^+^ T cells generated in hu-mice were functional. We immunized the hu-mice with MART-1 peptides emulsified by complete Freud's adjuvant and measured the immune response 3 weeks later. Tetramer^+^ CD8^+^ T cells showed a MART-1-specific response following immunization, as shown by antigen-specific expansion (Figure 2A), conversion from a naïve to effector/memory phenotype (i.e., losing CCR7 and CD45RA expression; Figure 2B), and IFN-γ production (Figure 2C). In contrast to tetramer^+^ CD8^+^ T cells, tetramer^−^ CD8^+^ T cells showed no detectable changes in either phenotypes or function after immunization with MART-1 peptides. However, unlike CD8^+^ T cells, neither tetramer^+^ nor tetramer^−^ CD4^+^ T cells showed expansion (Figure 2D), conversion to an effector/memory phenotype (Figure 2E) or IFN-γ production (Figure 2F) following MART-1 peptide immunization, despite that the tetramer^+^ CD4^+^ T cells showed significant proliferation after *in vitro* stimulation with MART-1 peptides (Figure S3). These data indicate that tetramer^+^ CD8^+^, but not CD4^+^, T cells could mount MART-1-specific responses following *in vivo* peptide immunization.

### MART-1-specific human T cells developed in humanized mice induce efficient killing of melanoma cells in an antigen-specific manner, and retain their antigen-specific cytotoxicity after extensive *in vitro* expansion

**Figure 3:**
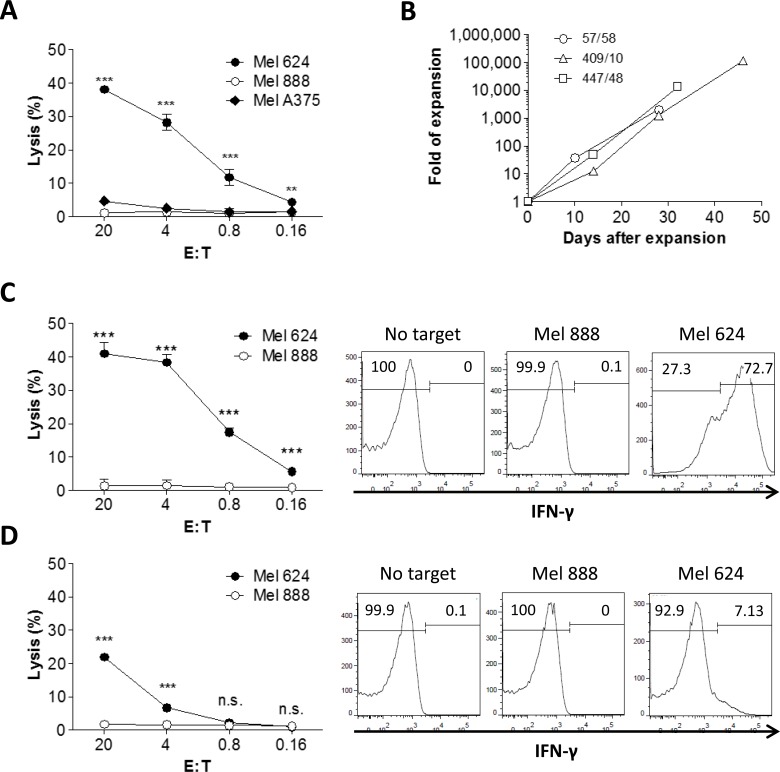
*In vitro* expansion and cytotoxicity of MART-1 TCR**+** T cells isolated from humanized mice **A.** Tetramer^+^ T cells were purified from spleen and bone marrow of hu-mice and their cytotoxicity against Mel 624 (HLA-A2^+^MART-1^+^), Mel 888 (HLA-A2^−^MART-1^+^), Mel A375 (HLA-A2^+^MART-1^−^) melanoma cells was measured after a short period (5 days) of *in vitro* stimulation with anti-huCD3/CD28 microbeads. **B.** *In vitro* expansion of tetramer^+^ T cells in the presence of OTK3, rhIL-12 and irradiated feeder cells. Tetramer^+^ T cells from 3 representative hu-mice are shown and data are presented as fold of expansion in cell numbers. **C.** and **D.** Tetramer^+^ CD8^+^ **C.** and CD4^+^ **D.** T cells sorted from *in vitro* expanded tetramer^+^ cells (shown in **B.**) were examined for cytotoxicity against melanoma cells using ^51^Cr release assay (left panel) and for IFN-γ production after cocultured with melanoma cells by flow cytometry (right panel). *, *p* < 0.05; **, *p* < 0.01; ***, *p* < 0.001; n.s., not significant.

We then measured the killing of melanoma cells by tetramer^+^ T cells isolated from hu-mice. Tetramer^+^ human T cells purified from hu-mice were stimulated for 5 days with anti-huCD3/CD28 microbeads, and their cytotoxicity against melanoma cells was assessed by ^51^Cr-release assay. As shown in Figure 3A, tetramer^+^ T cells induced significant killing of HLA-A2^+^MART-1^+^ melanoma cells (Mel 624), providing further evidence that human T cells developing from the engineered CD34^+^ cells in hu-mice were functional. Moreover, these T cells failed to produce cytotoxicity against HLA-A2^−^MART-1^+^ (Mel 888) and HLA-A2^+^MART-1^−^ (Mel A375) melanoma cells (Figure 3A), demonstrating an HLA-A2-restricted, MART-1-specific cytotoxic activity of tetramer^+^ T cells developed in the hu-mice.

Tetramer^+^ human T cells were prepared from the hu-mice and expanded for 4-7 weeks (approximately 2×10^3^ to 1×10^5^ fold increase in number) in media containing anti-huCD3 (OTK3), rhIL-2 and feeder cells (Figure 3B). CD8^+^ and CD4^+^ T cells were then sorted out from the expanded tetramer^+^ T cells and evaluated for antitumor activity. As shown in Figure 3C (left panel), tetramer^+^ CD8 T cells mediated efficient killing of HLA-A2^+^MART-1^+^ (Mel 624), but not HLA-A2^−^MART-1^+^ (Mel 888), melanoma cells. Furthermore, IFN-γ secretion was detected in tetramer^+^ CD8^+^ T cells cocultured with HLA-A2^+^MART-1^+^ melanoma (Mel 624) cells, but not in those cocultured with HLA-A2^−^MART-1^+^ melanoma (Mel 888) cells (Figure 3C, right panel). Similarly, high levels of granzyme B were detected in tetramer^+^ CD8^+^ T cells cocultured with Mel 624 cells, but not in those cocultured with Mel 888 cells (Figure S4). However, perforin production was not induced in tetramer^+^ CD8^+^ T cells after stimulation with either Mel 624 or Mel 888 cells (Figure S4), suggesting that perforin is not a major effector molecule for these T cells. Although to a markedly lower extend compared to tetramer^+^ CD8^+^ T cells, tetramer^+^ CD4^+^ T cells were also capable of mediating specific cytotoxicity and producing IFN-γ when cocultured with Mel 624, but not Mel 888, melanoma cells (Figure 3D). Taken together, these data demonstrate that tetramer^+^ human T cells (especially CD8 T cells) that developed in hu-mice are capable of killing melanoma cells in an HLA-A2/MART-1 peptide complex-dependent manner. Furthermore, these tetramer^+^ T cells can be substantially expanded *in vitro* without significantly compromising their cytotoxic activity.

### Adoptive immunotherapy using *in vitro*-expanded MART-1-specific T cells generated in humanized mice

**Figure 4:**
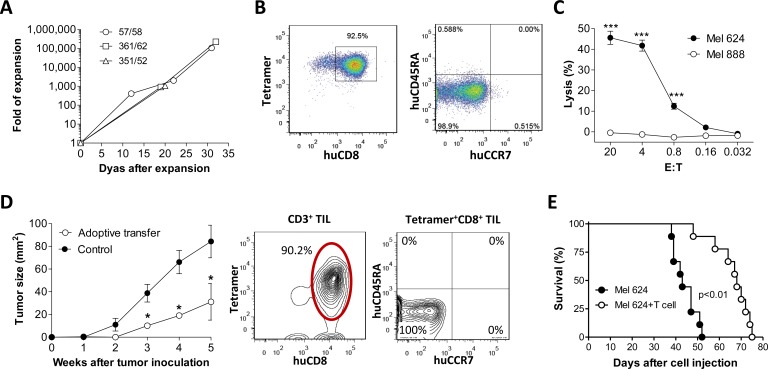
Antitumor effect by *in vitro* expanded MART-1-specific human CD8 T cells from humanized mice Tetramer^+^ CD8^+^ T cells purified from hu-mice (*n* = 3) were expanded in cultures as shown in Fig 3b **A.**-**C.**, and the anti-melanoma activity of *in vitro* expanded CD8^+^ T cells were assessed by adoptive transfer into melanoma-bearing recipients **D.**-**E. A.** Human T cell expansion in cultures at the indicated time points. **B.** Expression of MART-1-specific TCR, CD8, CD45RA and CCR7 on expanded human T cells. **C.** Cytotoxicity of the expanded human CD8^+^ T cells against melanoma cells. **D.** *Left panel*, tumor burden in mice receiving 1×10^6^ of Mel 624 (HLA-A2^+^MART-1^+^; s.c.) cells with or without (control) adoptive transfer of *in vitro*-expanded tetramer^+^ CD8 T cells (1×10^7^ per mouse ; *n* = 5 per group); *Right panel*, phenotypic analysis of CD3^+^ TILs at week 5 post-transfer of *in vitro*-expanded tetramer^+^ CD8 T cells. **E.** Survival of mice that received 2×10^5^ Mel 624 cells (i.v.) with or without adoptive transfer of 1×10^7^ *in vitro*-expanded tetramer^+^ CD8 T cells (*n* = 9 per group). *, *p* < 0.05; **, *p* < 0.01; ***, *p* < 0.001.

Tetramer^+^ CD8^+^ T cells were sorted out from hu-mouse splenocytes and expanded *in vitro* for approximately 30 days (Figure 4A). At the end of expansion, the majority of expanded cells were tetramer^+^ CD8^+^ T cells with a effector/memory (i.e., CCR7^−^CD45RA^−^) phenotype (Figure 4B), and capable of mediating efficient killing of HLA-A2^+^MART-1^+^ (Mel 624) melanoma cells (Figure 4C). We then assessed the antitumor activity of the expanded MART-1-specific CD8 T cells in melanoma-bearing mice. In NSG mice that were subcutaneously inoculated with Mel 624 melanoma cells (1×10^6^ per mouse), tumor growth was significantly inhibited by adoptive transfer of *in vitro* expanded tetramer^+^ CD8^+^ T cells (Figure 4D, left panel). Flow cytometric analysis revealed that most TILs were tetramer^+^ CD8^+^ T cells with a CD45RA^−^CCR7^−^ effector/memory phenotype (Figure 4D, right panel). Adoptive transfer of *in vitro* expanded tetramer^+^ CD8 T cells also mediated a significant protection against metastatic melanoma and markedly prolonged the survival in mice that were injected i.v. with Mel 624 melanoma cells (2×10^5^ per mouse; Figure 4E). These data provide direct *in vivo* evidence that tetramer^+^ CD8^+^ T cells developed in hu-mice are capable of infiltrating into tumors and killing tumor cells after adoptive transfer into tumor-bearing recipients.

### IL-15 promotes survival and antitumor responses of *in vitro* expanded MART-1 TCR^+^ T cells

**Figure 5:**
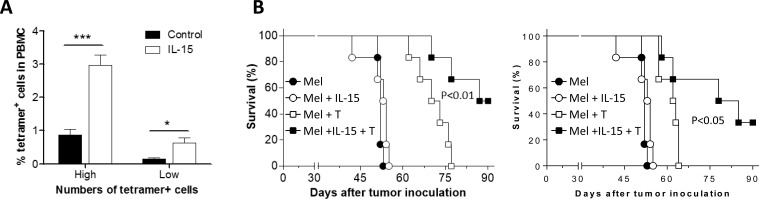
Improved antitumor effect by *in vitro* expanded MART-1-specific human CD8 T cells in IL-15-treated mice **A.** Survival of adoptively transferred *in vitro*-expanded tetramer^+^ CD8^+^ T cells (T cells were expanded as described in Figure 4A) in mice with or without IL-15 treatment at day 7 post-cell transfer (‘High’ and ‘Low’ represent mice receiving 1×10^7^ or 5×10^6^ tetramer^+^ CD8^+^ T cells per mouse, respectively; *n* = 6 per group). **B.** Survival of mice that received 2×10^5^ Mel 624 cells (i.v.) alone or together with 1×10^7^ (left) or 5×10^6^ (right) *in vitro*-expanded tetramer^+^ CD8 T cells with or without IL-15 treatment (*n* = 6 per group).*, *p* < 0.05; **, *p* < 0.01; ***, *p* < 0.001.

We then assessed the effect of IL-15 on the survival and antitumor activity of adoptively transferred tetramer^+^ T cells in a metastatic melanoma model, in which mice were injected i.v. with Mel 624 melanoma cells (2×10^5^ per mouse). IL-15 treatment was given by hydrodynamic injection of IL-15-expressing plasmids [12] one day prior to T cell transfer. At week 1 post-T cell transfer, the ratio of tetramer^+^ CD8^+^ T cells in peripheral blood was significantly (approximately 3-fold) greater in IL-15-treated than in the control group (Figure 5A). Importantly, this increase in tetramer^+^ CD8^+^ T cells in IL-15-treated mice was associated with a significantly enhanced antitumor response. As shown in Figure 5B, adoptive transfer of tetramer^+^ CD8^+^ T cells was significantly more effective in prolonging survival in IL-15-treated than in control mice bearing metastatic melanoma. These data indicate that IL-15 treatment has the potential to improve both the survival and antitumor activity of tumor antigen-specific CTLs. Because treatment with IL-15 alone did not induce detectable antitumor responses (Figure 5B), we conclude that the enhancement of antitumor effects by IL-15 was mediated by the adoptively transferred tetramer^+^ T cells.

### A short treatment of the expanded MART-1 TCR^+^ T cells with rapamycin immediately prior to adoptive therapy leads to markedly improved protection against metastatic melanoma

**Figure 6:**
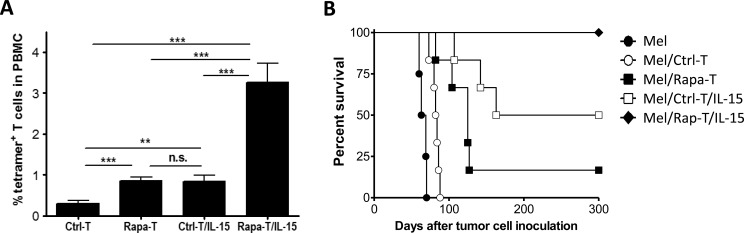
A short *in vitro* treatment with rapamycin significantly improves the survival and antitumor activity of *in vitro* expanded tetramer^**+**^ CD8**+** T cells **A.** Survival of adoptively transferred rapamycin-treated (Rap-T) and non-treated (Ctrl-T) tetramer^+^ CD8^+^ T cells at day 7 in mice with or without IL-15 treatment. **B.** Survival of IL-15-treated and non-treated mice that received injection i.v. of 1×10^5^ Mel 624 cells plus 5×10^6^ Ctrl-T (Mel/Ctrl-T and Mel/Ctrl-T/IL-15) or Rapa-T (Mel/Rapa-T and Mel/Rapa-T/IL-15) cells (*n* = 6 per group), and non-treated mice receiving 1×10^5^ Mel 624 cells only (Mel; *n* = 4). **, *p* < 0.01; ***, *p* < 0.001; n.s., not significant

We further explore the potential of treatment with rapamycin prior to adoptive transfer to improve the survival and antitumor activity of tumor antigen-specific T cells. *In vitro* expanded tetramer^+^ CD8^+^ T cells were cultured with rapamycin (100nM) for 3 days immediately before transfer into IL-15-treated or non-treated melanoma-bearing recipients that were injected i.v. with Mel 624 melanoma cells (1×10^5^ per mouse). A short treatment with rapamycin significantly improved T cell survival in both non-treated and IL-15-treated recipients (Figure 6A). In the recipients without IL-15 treatment, the percentage of rapamycin-treated tetramer^+^ CD8^+^ T (Rapa-T) cells measured at week 1 (0.86±0.09%) was significantly (approximately 3 fold) greater than that of non-treated tetramer^+^ T (Ctrl-T) cells (0.30±0.06%; Figure 6A). Treatment of the recipients with IL-15 synergistically enhanced the effect of rapamycin in improving T cell survival. The percentage of surviving Rapa-T cells at week 1 in IL-15-treated mice (Rapa-T/IL-15; 3.26±0.46%) was more than 10-fold and about 4-fold greater than that of Ctrl-T and Rapa-T cells, respectively, in mice that did not receive IL-15 treatment, and approximately 4-fold greater than that of Ctrl-T cells in IL-15-treated mice (Ctrl-T/IL-15; 0.84±0.14%; Figure 6A).

Rapa-T cells also mediated significantly stronger antitumor responses than Ctrl-T cells. Consistent with the results shown in Figure 5B, adoptive transfer of Ctrl-T cells induced significant protection against metastatic melanoma, which was further enhanced by IL-15 treatment (Figure 6B). Compared to Ctrl-T cells, Rapa-T cells were significantly more effective in eradicating melanoma, leading to improved survival rates in mice with metastatic melanoma (Figure 6B). Notably, a powerful antitumor response leading to 100% survival for more than 300 days was seen in IL-15-treated mice infused with Rapa-T cells, while most of the mice receiving Rapa-T cells without IL-15 and half of the IL-15-treated mice receiving Ctrl-T cells succumbed to metastatic melanoma. All surviving mice were sacrificed at day 320 post-infusion and none showed detectable tumors at autopsy.

## DISCUSSION

In this study, we developed a hu-mouse model that permits efficient generation of tumor antigen-specific human T cells from genetically engineered CD34^+^ cells. Using this model in combination with *in vitro* cell expansion, we were able to produce large quantities of melanoma antigen MART-1-specific T cells with antigen-specific antitumor activity. This approach provides a practical means of producing potentially unlimited source of tumor-specific human T cells, which can be used in the development and testing of new cancer immunotherapy protocols in the preclinical settings. The recent development of “personalized immune (PI)” hu-mice demonstrates the feasibility of generating patient-specific human T cells [13]. Thus, the combination of the strategy described in the current study with the “PI” hu-mouse model may permit the production of tumor-reactive human T cells for preclinical exploration of individualized cancer immunotherapy.

Clinical trials using autologous peripheral lymphocytes genetically engineered by retroviral vector containing tumor antigen specific TCR gene have shown objective cancer regression in 10-30% patients with metastatic melanoma [14-16]. However, a challenge for this strategy is to prevent misparing of the endogenous TCR subunits with introduced TCR chains in the genetically-manipulated T cells, which may potentially alter TCR specificity leading to not only the loss of anti-tumor responses, but also formation of autoreactive T cells [17-19]. Unlike genetically engineered mature T cells, MART-1-TCR^+^ human T cells in the hu-mouse are developed from TCR gene-transduced hematopoietic stem cells, so that most of these cells should express only the tumor antigen-specific TCR.

As expected, the majority of human T cells expressing the HLA-A2-restricted MART-1-TCR^+^ developing in hu-mice expressed CD8. However, these hu-mice also had a significant population of tetramer^+^ CD4^+^ T cells including FoxP3^+^CD4^+^ Tregs (Figure S5). Similar to our results, the presence of CD4^+^ [20] and CD4^−^CD8^−^ [21] human T cells that recognize melanoma antigens in an HLA class I-restricted manner was also found in humans. Previous studies on the ability of MHC class I-restricted CD4 T cells to generate antitumor effects have been contradictory [22-25]. A recent study showed that the antitumor efficacy of CD4^+^ T cells expressing MHC class I-restricted TCR depends on TCR affinity, and only those with high affinity TCR may mediate CD8-independent cytotoxicity [26]. In the present study, we showed that, although to a significantly lower extent than tetramer^+^ CD8^+^ T cells, tetramer^+^ CD4^+^ T cells developing in the hu-mice were also functional and capable of mediating antigen-specific responses (i.e., cytotoxicity and IFN-γ production) *in vitro* when cocultured with the target cells. However, we failed to detect antigen-specific responses in tetramer^+^ CD4^+^ T cells from MART-1 peptide-immunized hu-mice, despite that tetramer^+^ CD8^+^ T cells from the same hu-mice responded robustly to immunization. Clearly, further studies are needed to determine whether the tetramer^+^ CD4^+^ T cells may provide help to CD8 T cells expressing the same TCR. Nonetheless, our results implicate that *in vitro* analysis may not accurately predicate the *in vivo* antitumor effect of tumor antigens-specific T cells.

IL-15 is an immune regulatory cytokine with broad activities that include inducing differentiation and proliferation of T, B and NK cells, enhancing the cytolytic activity of CD8^+^ T cells and contributing to long term survival of memory T cells [27]. The potential of IL-15 to promote the survival and antitumor activity of adoptively transferred tumor-specific CD8^+^ T cells has been demonstrated in mice [28, 29], but remains poorly understood in humans. In the present study, we observed that *in vivo* treatment with IL-15 dramatically promoted the survival and/or proliferation of activated MART-1-TCR^+^ CD8 T cells, which was associated with a significantly increased antitumor effect, in melanoma-bearing hosts. The data support the idea of using IL-15 to enhance antitumor responses of adoptive T-cell transfer therapy, and a clinical trial launched recently to test the effectiveness of TIL administration combined with IL-15 in metastatic melanoma [27].

Another novel observation made in this study is that IL-15 administration combined with infusion of rapamycin-treated MART-1-TCR^+^ human CD8^+^ T cells produced a strong synergistic effect in promoting the survival and antitumor responses of adoptively transferred tumor antigen-specific human T cells. It has been shown that inhibition of mTORC1 by rapamycin promotes memory CD8^+^ T cell generation and function [30, 31] through down-regulation of T-bet and upregulation of Eomes in CD8^+^ T cells [32]. Using TCR transgenic mouse models, it was recently reported that a short course of injection with rapamycin enhances vaccine-induced CD8^+^ T cell memory and antitumor responses in mice [33, 34]. In this study, we observed that a brief culture of human CD8^+^ T cells with rapamycin significantly improved their survival and antitumor responses in melanoma-bearing recipients, particularly in mice treated IL-15. Since enhanced CD8^+^ T cell memory responses by mTOR inhibition is independent of IL-15 [32, 33], rapamycin-treated CD8 T cells should retain the ability to respond to the survival signaling provided by IL-15, which may explain why IL-15 and rapamycin can act synergistically to improve the survival and antitumor responses of tumor antigen-specific CD8^+^ T cells. The combinatorial treatment of the tumor-reactive CD8 T cells with rapamycin and of the recipient with IL-15 may provide a novel protocol for potentiating adoptive T cell immunotherapy.

## MATERIALS AND METHODS

### Animals and human tissues and cells

NOD.Cg-Prkdc^scid^ Il2rg^tm1Wjl^/SzJ (NOD/SCID/c^−/−^ or NSG) mice were purchased from the Jackson Laboratory (Bar Harbor, ME), housed in a specific pathogen-free micro-isolator environment and used in experiments at 6 to 8 weeks of age. Human fetal thymus and liver tissues of gestational age of 17 to 20 weeks were obtained from Advanced Bioscience Resource (Alameda, CA). Protocols involving the use of discarded human tissues and animals were approved by the Institutional Review Board (IRB) and Institutional Animal Care and Use Committee (IACUC) of Columbia University Medical Center (New York, NY).

### Humanized mouse preparation

NSG mice were conditioned with sublethal (2 Gy) total body irradiation, and received lentiviral vector transduced human CD34^+^ FLCs (2×10^5^/mouse, i.v.) with a cryopreservation treated FTHY fragment measuring about 1mm^3^ (under the recipient kidney capsule) from the same fetal donor, as previously described [9, 10]. CD34^+^ FLCs were isolated by a magnetic-activated cell sorter (MACS) separation system using anti-CD34 microbeads (Miltenyi Biotec, Auburn, CA). Levels of human hematopoietic cells in humanized mice were determined by flow cytometric (FCM) analysis using various combinations of the following mAbs: anti-human CD45, CD19, CD3, CD4, CD8, CD45RA, CCR7; anti-mouse CD45 and Ter119; and isotype control mAbs (all antibodies were purchased from BD PharMingen, San Diego, CA). MART-1-TCR^+^ T cells were identified by HLA-A*0201/MART-1 (ELAGIGILTV) Tetramer (Beckman Coulter Immunotech). Mononuclear cells were prepared using density gradient centrifugation with Histopaque 1077 (Sigma-Aldrich, St. Louis, MO). Analysis was performed on a FACSCanto or LSR II (Becton Dickinson, Mountain View, CA). Dead cells were excluded from the analysis by gating out lower forward scatter and high propidium iodide-retaining cells.

### Lentiviral production and human CD34^+^ FLC transduction

Pseudotyped lentiviral vectors were produced by transfection using Lipofectamine 2000 (Invitrogen, San Diego, CA) of 293FT cells in 10-cm plates. a 4-plasmid system consisting of the transfer vector (MART-1 antigen specific TCR, DMF5 clone; 10μg) and 3 packaging plasmids (VSV-G 3.6μg, pMDLg/pRRE 6.7 μg, and pRSV-Rev 6.7 μg) was used for MART-1 TCR-lentivirus production. The supernatant was collected 48 hour post-transfection and concentrated by ultracentrifugation at 50,000 g for 2 hours. Lentiviruses were stored at −80°C until use. Human CD34^+^ FLCs were stimulated overnight in media containing 50ng/mL rhSCF (R&D, Minneapolis, MN), 50ng/mL Flt3L (ebioscience, San Diego, CA), 25 ng/mL TPO (R&D), and 10ng/mL IL-3 (R&D), in a 24-well plate pre-coated with retronectin (Takara Bio Inc), followed by transduction with lentiviral vectors for 12 hours. Cells were washed twice and intravenously injected into sub-lethal irradiated mice as described above.

### MART-1 peptide immunization

MART-1 peptides (ELAGIGILTV, Biosynthesis Inc, Lewisville, Texas) were emulsified with Freud's Complete Adjuvant (CFA) with 1:1 ratio and subcutaneously injected into humanized mice. Immunized mice were analyzed for MART-1-specific responses 3 weeks after immunization.

### CTL assay

Purified MART-1 tetramer^+^ human T cells were co-incubated with ^51^Cr-labeled melanoma cells at the indicated effector:target (E:T) ratios in 96-well plates at 37°C for 4-6 hours. Lysis of tumor cells was measured by ^51^Cr release in the supernatant counted using a Perkin Elmer 1450 microbeta liquid scintillation & luminescence counter. Specific lysis (%) of target cells was calculated as: = [(experimental release (cpm) - spontaneous release (cpm)]/[(maximum release (cpm) - spontaneous release (cpm)] ×100%.

### *In vitro* expansion of MART-1 antigen specific T cells

MART-1 tetramer^+^ T cells were purified from lymphoid organs of humanized mice by cell sorting using an Influx cell sorter (BD Biosciences) and expanded *in vitro* using a previously described protocol with some modification [35, 36]. Briefly, purified T cells were stimulated with pooled allogeneic PBMCs (1.5×10^7^/mL, 35 Gy irradiated) and Epstein-Barr virus-transformed lymphoblastoid cell lines (EBV-LCL, 4.3×10^5^/mL, 60 Gy irradiated), 30 ng/mL anti-huCD3 (OKT3), and 100U/mL recombinant human IL-2 (rhIL-2) in RMPI 1640 medium supplemented with 10% human AB serum. One third of the culture supernatant was replaced with fresh media containing 300U/mL rhIL-2 every 2-3 days. The cells were restimulated with anti-human CD3 and freshly irradiated feeder cells (i.e., allogeneic PBMCs and EBV-LCL) every 2 weeks.

### Assessment of antitumor effects of *in vitro*-expanded MART-1 TCR^+^ T cells in melanoma-bearing mice

*In vitro* expanded CD8 MART-1 TCR^+^ T cells were adoptively transferred (i.v.) into NSG mice that were inoculated with Mel 624 cells (s.c. or i.v.) on the same day, and the antitumor effect was evaluated by measuring tumor mass or recipient survival time in mice receiving s.c. and i.v. injection of melanoma cells, respectively. Tumor mass was measured using fine calipers (Marathon, Richmond Hill, Canada) and calculated by the product of the two largest perpendicular diameters a×b (mm^2^), as previously described [6]. To assess the effect of IL-15 on antitumor responses, some recipient mice were given hydrodynamic injection (50μg/2mL/mouse *via* tail vein) of plasmid containing rhIL-15 gene one day prior to injection of melanoma cells, as previously described [12]. In some experiments, rapamycin (100nM; Sigma Aldrich; St. Louis, Missouri) was added into the T cell expansion culture for 3 days immediately before adoptive transfer.

### Statistical analysis

The level of significant differences in group means was determined by the student's *t* test. Graft survival data were presented as Kaplan-Meier survival curves and differences between groups were analyzed by logrank test using GraphPad Prism (San Diego, CA). All statistical analysis was performed using Prism 4 (GraphPad Software, San Diego, CA). A *p* value of ≤ .05 was considered significant in all analyses herein.

## SUPPLEMENTARY TABLE AND FIGURES


