# Supplementary material for: Human melanoma immunotherapy using tumor antigen-specific T cells generated in humanized mice
Source: Oncotarget. 2016 Jan 27;7(6):6448–59. doi: 10.18632/oncotarget.7044 (PMC4872726; doi:10.18632/oncotarget.7044)
Supplement: Supplementary file 1 [file oncotarget-07-6448-s001.pdf]

Human melanoma immunotherapy using tumor antigen-specific T cells generated in humanized mice

Supplementary Material

Table S1. Numbers of tetramer$^+$ CD8 T cells in hu-mouse spleens

**Hu-mouse**[1] (Eartag)	**Time of analysis** (Weeks post-Tx)	**Treatment**[2]	**Tetramer$^+$ CD8 cells** ($x10^6$/spleen)
359/360	19	PBS	1.1
351/352	19	MART-1	9.3
361/362	20	PBS	1.2
355/356	21	PBS	1.8
353/354	21	MART-1	8.0
171/172	34	None	6.0
173/174	34	None	9.8
175/176	34	None	12.0

[1] Hu-mice from two experiments were analyzed: first 5 mice were from one experiment and the rest were from another one.

[2] Hu-mice were immunized with PBS or MART-1 peptides 3 weeks prior to analysis, or received no treatment.

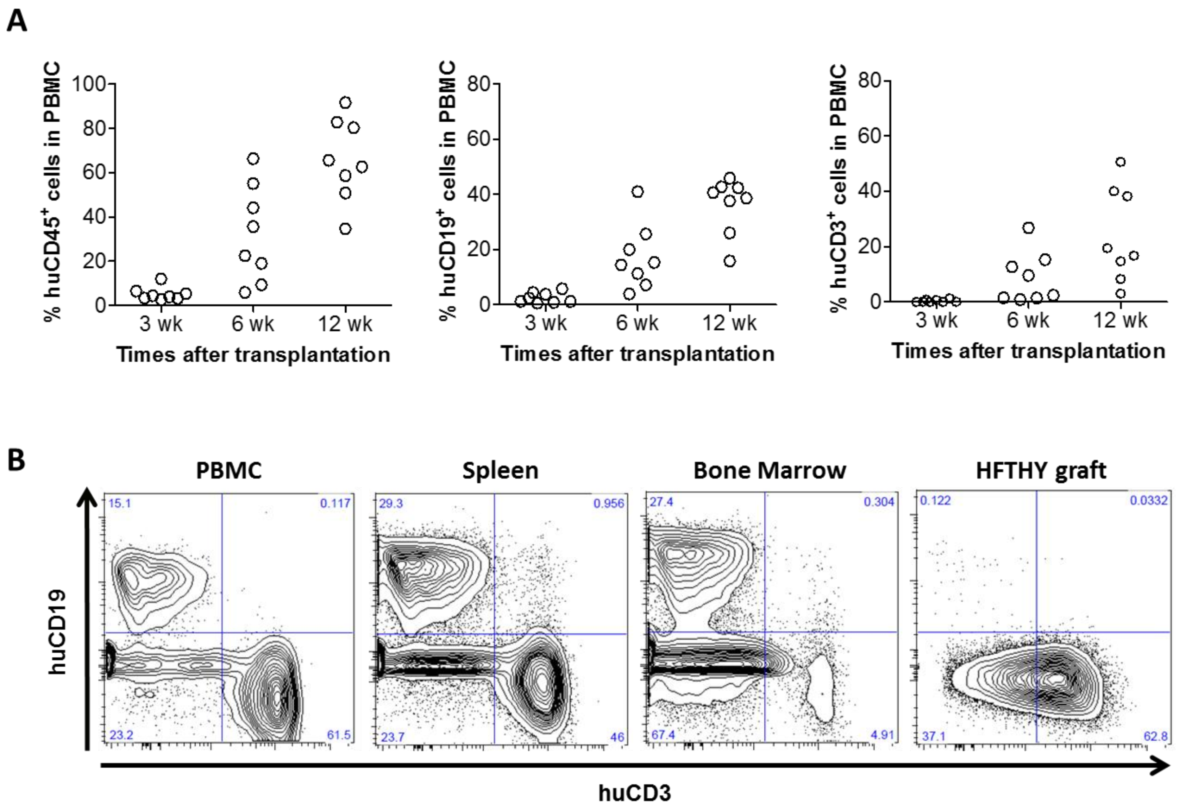

Fig. S1. Human lymphohematopoietic cell reconstitution in humanized mice. Flow cytometric profiles showing multilineages of human lymphohematopoietic cell reconstitution in blood at the indicated times (**A**), and in various tissues prepared from a representative hu-mouse 22 weeks (**B**) after transplantation of human FTHY and $CD34^+$ FLCs virally-transduced with MART-1-specific TCR gene.

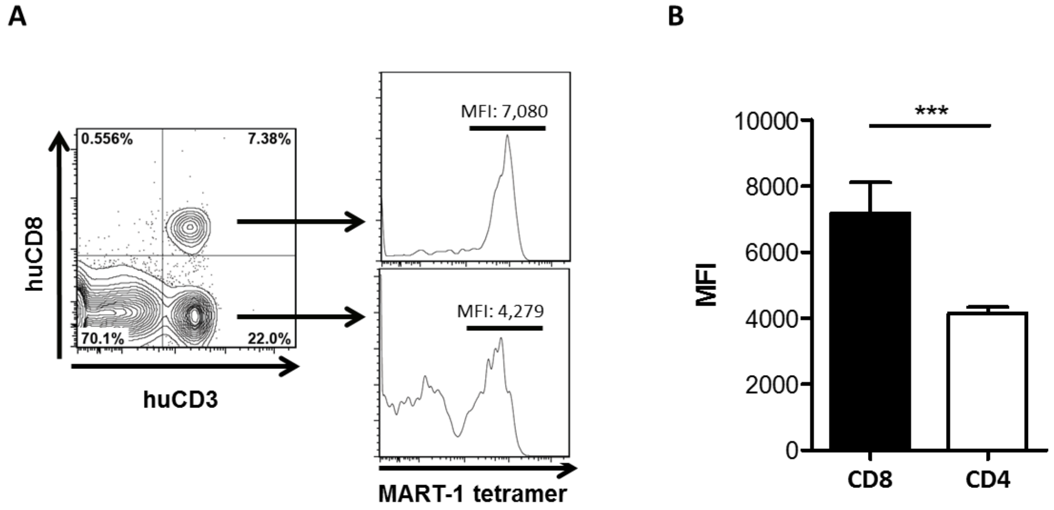

Fig. S2. CD4 T cells express a lower level of MART-1-specific TCR than CD8 T cells. (**A**) Representative flow cytometric profiles showing reconstitution of MART-1-specific TCR^+ cells in peripheral blood $CD8^+$ and $CD8^-$ (i.e., $CD4^+$ confirmed by staining with anti-CD4 mAb) and fluorescence intensity of MART-1 tetramer staining 14 weeks after humanization. (**B**) Median fluorescence intensity (MFI; mean±SEMs; n=6) of MART tetramer staining in CD8+ and CD4+ T cells. ***, $p<0.001$.

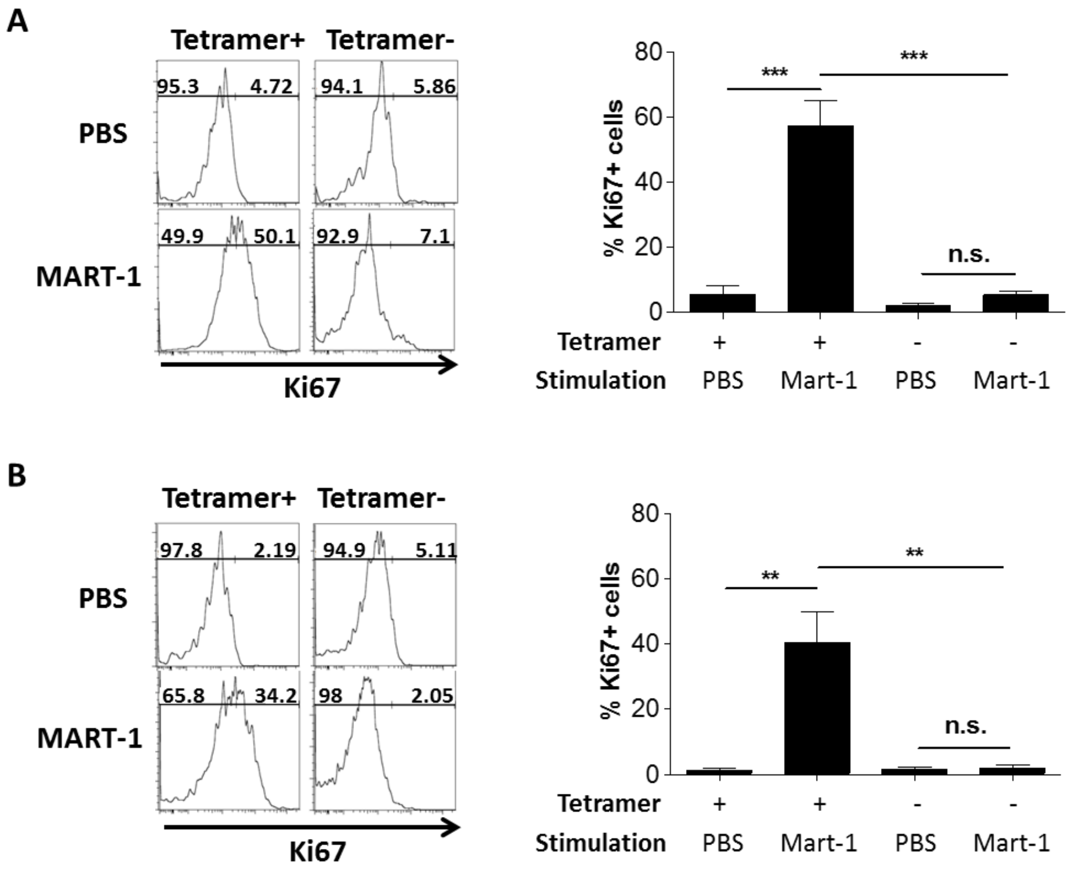

Fig. S3. Proliferation of tetramer$^+$ T cells in response to MART-1 stimulation. Spleen cells harvested from hu-mice were incubated with MART-1 peptides (10µg/mL) or PBS as control for 3 days. Proliferation of CD8$^+$ (**A**) or CD4$^+$ (**B**) T cells was then determined by measuring Ki67 expression. N=7 per group. *, P<0.05; **, P<0.01; ***, P<0.001; n.s., not significant.

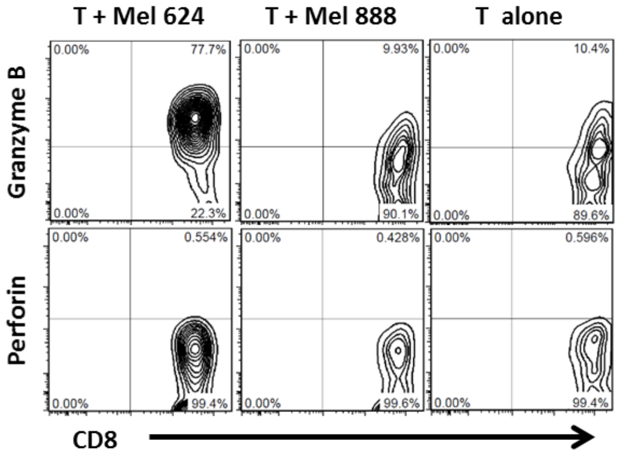

Fig. S4. Expression of granzyme B and perforin in MART-1-TCR$^+$ human CD8 T cells following stimulation with melanoma cells. MART-1-TCR$^+$ human CD8 T cells were cultured alone or stimulated with Mel 624 (HLA-A2$^+$MART-1$^+$) or Mel 888 (HLA-A2$^-$MART-1$^-$) melanoma cells for 4-6 hours, and the secretion of granzyme B and perforin was determined by flow cytometric analysis. Results from a representative of two independent experiments are shown.

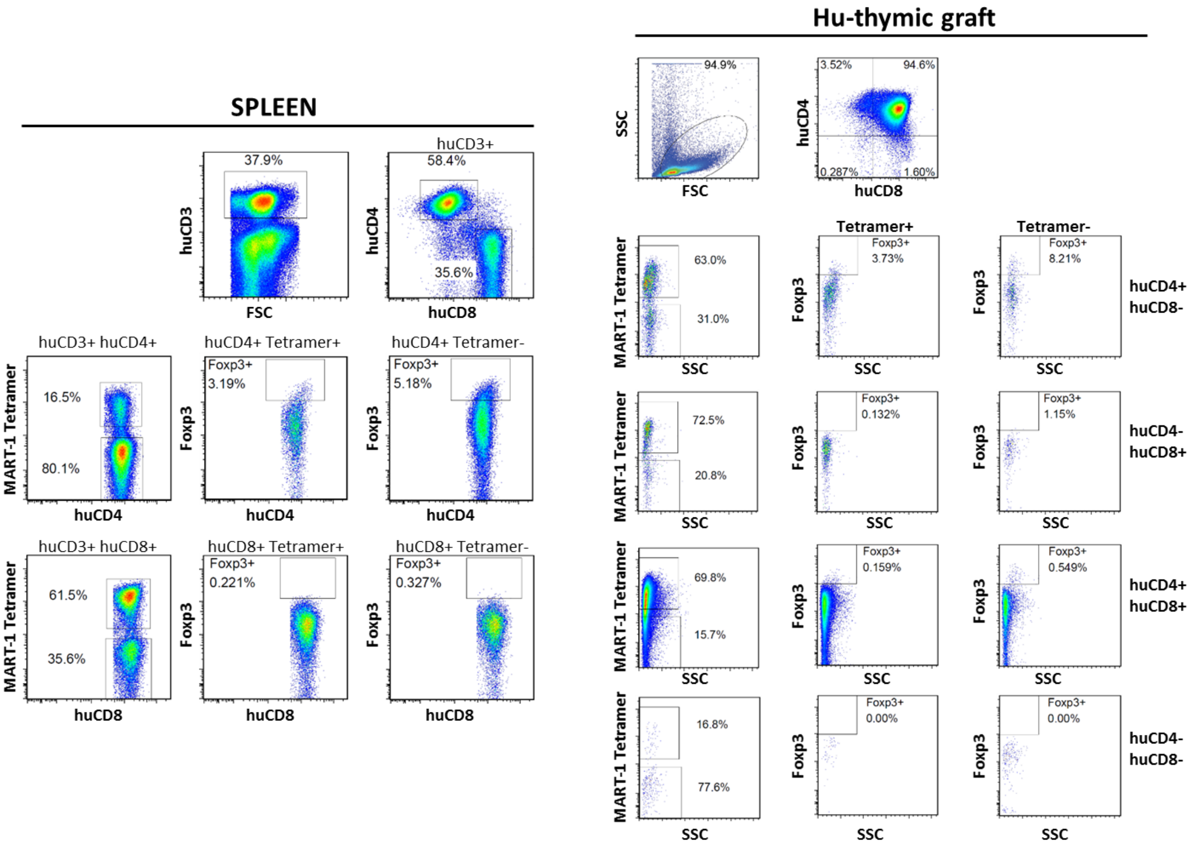

Fig. S5. MART-1-TCR$^+$ human regulatory T cell development in humanized mice. FACS analysis was performed 17 weeks after human thymus/CD34$^+$ cell transplantation. Shown are representative flow cytometric profiles of Foxp3 expression in MART-1 TCR$^+$ or MART-1 TCR$^-$ human T cells in the spleen (A) or thymocyte subsets in the human thymic graft (B).
